# A Pathophysiological Approach to Reduce Peritumoral Edema with Gamma Knife Radiosurgery for Large Incidental Meningiomas

**DOI:** 10.3390/life12111683

**Published:** 2022-10-23

**Authors:** Cheng-Siu Chang, Cheng-Wei Huang, Hsi-Hsien Chou, Hsien-Tang Tu, Ming-Tsung Lee, Chuan-Fu Huang

**Affiliations:** 1 Department of Neurosurgery, Chung-Shan Medical University Hospital, Taichung 402, Taiwan; 2 School of Medicine, Chung-Shan Medical University, Taichung 402, Taiwan; 3 Department of Hospital Medicine, Kaiser Permanente Los Angeles Medical Center, Los Angeles, CA 90027, USA; 4 Department of Clinical Science, Kaiser Permanente Bernard J. Tyson School of Medicine, Pasadena, CA 91101, USA; 5 Gamma Knife Center, Chang Bing Show Chwan Memorial Hospital, Changhua 505, Taiwan; 6 National Center for Geriatrics and Welfare Research, National Health Research Institutes, Yunlin 632, Taiwan; 7 Department of Nursing, Hungkuang University, Taichung 433, Taiwan

**Keywords:** peritumoral edema, gamma knife, radiosurgery, stereotactic radiosurgery, incidental meningioma, meningioma

## Abstract

Background: Peritumoral edema may be a prohibitive side effect in treating large incidental meningiomas with stereotactic radiosurgery. An approach that limits peritumoral edema and achieves tumor control with SRS would be an attractive management option for large incidental meningiomas. Methods: This is a retrospective cohort study of patients with large incidental meningiomas (≥2 mL in volume and/or 2 cm in diameter) treated with gamma knife radiosurgery (GKRS) between 2000 and 2019 in Taiwan and followed up for 5 years. The outcomes of a pathophysiological approach targeting the dural feeding artery site with a higher marginal dose (18–20 Gy) to enhance vascular damage and the parenchymal margin of the tumor with a lower dose (9–11 Gy) to reduce parenchymal damage were compared with those of a conventional approach targeting the tumor center with a higher dose and tumor margin with a lower dose (12–14 Gy). Results: A total of 53 incidental meningiomas were identified, of which 23 (43.4%) were treated with a pathophysiological approach (4 cases underwent a two-stage approach) and 30 (56.7%) were treated with a conventional approach. During a median follow-up of 3.5 (range 1–5) years, tumor control was achieved in 19 (100%) incidental meningiomas that underwent a single-stage pathophysiological approach compared with 29 (96.7%) incidental meningiomas that underwent a conventional approach (log-rank test: *p* = 0.426). Peritumoral edema developed in zero (0%) incidental meningiomas that underwent a single stage pathophysiological approach compared to seven (23.3%) incidental meningiomas that underwent a conventional approach (log-rank test: *p* = 0.023). Conclusions: Treatment of large incidental meningiomas with a pathophysiological approach with GKRS achieves similar rates of tumor control and reduces the risk of peritumoral edema. GKRS with a pathophysiological approach may be a reasonable management strategy for large incidental meningiomas.

## 1. Introduction

Incidental or asymptomatic meningiomas are increasingly diagnosed due to the widespread use of brain imaging, such as magnetic resonance imaging. Treatment options include surveillance, surgery, and stereotactic radiosurgery. Regular observation is the simplest method, but the persistent growth rate is relatively high for patients with a longer period of survey [1,2]. Hashiba et al. reported 42 tumors exhibited growth in 70 patients with incidentally discovered meningiomas who underwent follow-up for longer than 1 year [1]. Surgery, on the other hand, is combined with postoperative morbidity and mortality, which is not negligible, especially in asymptomatic patients with a more advanced age [1,3]. Reinert et al. showed that the overall risk of permanent neurological morbidity was 4.9% in asymptomatic and 23.2% in symptomatic patients in a total of 201 patients, of whom 102 were asymptomatic and 99 were symptomatic [3]. Stereotactic radiosurgery has been successfully used as both a primary and an adjuvant treatment for incidental meningiomas given its minimal invasiveness and safety [2,4,5,6]. However, stereotactic radiosurgery can be associated with complications such as peritumoral edema. Peritumoral edema is a known complication of stereotactic radiosurgery, with a reported prevalence of 15–28% and associated neurological symptoms in 3–15% of intracranial meningioma cases [4,6,7,8,9,10,11,12,13].

The risk of peritumoral edema with treatment of incidental meningiomas increases with tumor size, among other factors [9,14]. This association may be explained by the higher margin doses used to treat larger tumors, which results in damage to the leptomeninges and brain cortex. The damaged leptomeninges allows for the direct transmission of humoral edema-promoting factor or edema fluid into the white matter, which causes vasogenic edema, so it is reasonable to lower the treatment dose of parenchymal margin to reduce the peritumoral edema [15,16]. On the other hand, meningiomas derive from the meningothelial cells of the arachnoid layer and grow based on the dura mater. The majority of tumors are predominantly supplied by meningeal vessels and cause a sunburst or spoke-wheel pattern; higher doses for dural sites to damage vascular tumors, and relatively lower doses for the parenchymal margin may help to maintain tumor control and reduce radiation side effects. Minimal side effects are required to justify pursuing treatment in incidental meningiomas, given that observation is a reasonable strategy with only a small risk of tumor progression [17]. Approaches to reduce peritumoral edema, if identified, would make stereotactic radiosurgery a more attractive management option for large incidental meningiomas as incidental meningiomas are increasingly diagnosed.

We hypothesized that a pathophysiological approach that irradiates the dural feeding artery(s) with a higher dose would enhance the radiobiologic effect and allow for a lower dose at the parenchymal margin while reducing the risk of peritumoral edema and achieving similar tumor control. In this paper, we describe our experience in treating large incidental meningiomas with GKRS and compare the effectiveness and safety of a pathophysiological approach with a conventional approach.

## 2. Methods

### 2.1. Study Design and Population

This is a retrospective cohort of patients with large incidental meningiomas (≥2 mL in volume and/or 2 cm in diameter) treated with GKRS between 2000 and 2019. Incidental meningioma was defined as a meningioma incidentally found on the MRI of patients who underwent imaging for an unrelated evaluation. The indication for treatment was tumor progression, defined as an increase in tumor size by >15% or growth in a functional area including the motor, the sensory, or the speech region, or the posterior fossa or the cavernous sinus. We excluded patients who had incidental meningioma with evidence of peritumoral edema at time of diagnosis given that they would not be candidates for GKRS due to the concern for edema aggravation.

Baseline characteristics, clinical, and radiological data were manually abstracted through chart review. The study was approved by our Institutional Review Board (IRB# 1090907) and informed consent was waived.

### 2.2. Treatment Approach and Exposure Variable

High-resolution stereotactic gadolinium-enhanced MRI was performed to determine target coordinates and dose planning. The Leksell stereotactic coordinate frame was applied after the patients received a local anesthetic.

**Figure 1:**
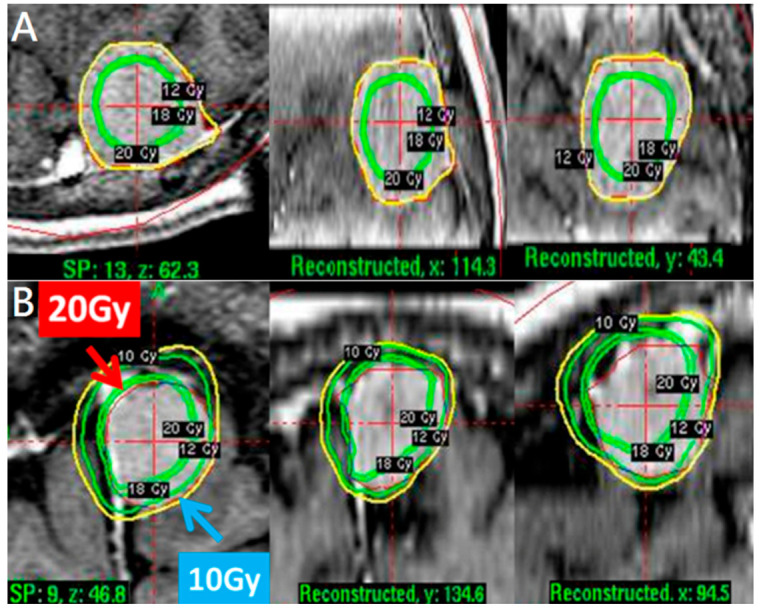
(**A**) A conventional approach with tumor marginal dose 12 Gy and higher dose at tumor center in axial, coronal, and sagittal views. A pathophysiological approach. (**B**) A tumor volume 3.7 mL treated with 20 Gy for dural site and 10 Gy for parenchymal margin in axial, coronal, and sagittal views.

Prior to 2014, we used a conventional approach that targeted the tumor center with a tumor margin with a peripheral dose (12–16 Gy) (Figure 1A). In 2014, we began to employ a pathophysiological approach to reduce peritumoral edema for large incidental meningiomas.

**Figure 2:**
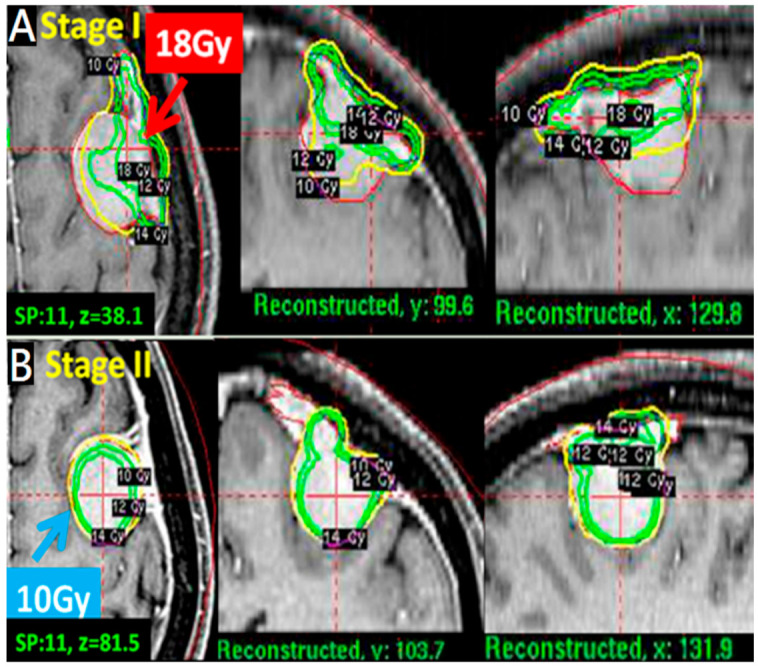
A pathophysiological approach for tumors >10 mL treated with two-stage strategy. (**A**): A convex tumor (11 mL); stage 1 with higher dose (18 Gy) first for dural attachment site to destroy blood supply entry point. (**B**) Stage 2 with lower dose (10 Gy) for parenchymal margin site in axial, coronal and sagittal views.

A pathophysiological approach targeted the dural feeding artery site with a higher dose (18–20 Gy) to enhance vascular damage and the parenchymal margin of the tumor with a lower dose (9–11 Gy) to reduce peritumoral edema (Figure 1B). The higher dose covered the whole dural margin to include all the feeding arteries, in the event that there were multiple feeding arteries. For especially large tumors > 10 mL in volume, treatments were staged, with stage 1 targeting the dural site, followed by stage 2 targeting the parenchymal margin at 6 months apart (Figure 2A,B).

### 2.3. Outcomes and Follow-Up Duration

The primary effectiveness outcome was tumor control, defined as tumor volume reduction (≥15% volume reduction) or stability (within 15% volume change) on the follow-up MRI. Treatment failure or tumor progression was defined by either an increase in tumor volume > 15% and/or the need for additional surgical management.

The primary safety outcome was peritumoral edema identified by magnetic resonance imaging. T2-weighted images were used to evaluate peritumoral edema before and after GKRS treatment. For images obtained before GKRS treatment, the presence or absence of peritumoral edema was identified. The edema index was defined as the ratio of peritumoral high-signal-intensity volume, including tumor volume on a T2-weighted MRI to tumor volume on a T1-weighted gadolinium-enhanced MRI. In each tumor, serial EIs were measured on follow-up MRIs after GKRS, and the relative edema indices were calculated from these values and normalized against the baseline edema index. Any neurological symptoms that matched with increased peritumoral edema on an MRI were regarded as symptomatic peritumoral edema.

All patients were followed for up to 5 years to prevent selection bias as a result of different follow-up periods in the two groups.

### 2.4. Statistical Analysis

Descriptive statistics are used to describe the series by treatment approach. Continuous variables are presented as median and range. Categorical variables are presented as absolute numbers and percentages (%). Continuous variables were compared using the Mann–Whitney U test and categorical variables were compared using Fisher’s exact test. A Kaplan–Meier (KM) analysis with a log-rank test was also performed to examine outcomes by treatment approach. For the KM analysis, we excluded the four cases that required a two-stage approach to minimize any confounding. introduced by difference in approach. For all comparisons, a *p*-value < 0.05 was considered statistically significant. All analyses were performed by IBM SPSS Statistics for Windows, version 24.0 (IBM Corp., Armonk, NY, USA).

## 3. Results

### 3.1. Baseline Characteristics

A total of 53 patients were included in this study. The median age was 64 years old (range: 33–87 years). Of the patients, 46 (86.8%) were women, and they also dominated in both groups (Table 1).

Tumor locations were divided into hemispheric and skull base location. Of all the tumors, 37 (69.8%) were in the hemispheric location, and 16 (30.2%) were skull base location. Of the 37 hemispheric tumors, 21 (21/37) were treated with a pathophysiological approach, of which 4 underwent a two-stage approach, and 16 (16/37) were treated with a conventional approach (largely treated prior to 2014). Of the 16 skull base tumors, only two cases were treated with a pathophysiological approach, while 14 were treated with conventional approach.

Of the 53 incidental meningiomas, 30 (56.7%) incidental meningiomas were treated with a conventional approach, and 23 (43.4%) incidental meningiomas were treated with a pathophysiological approach, of which 4 underwent a two-stage approach (Table 1). The median tumor volume was 3.5 (range: 2–27.2) mL. The tumor volume was significantly larger in incidental meningiomas treated with the pathophysiological approach compared with the conventional approach (median volume: 5.3 mL vs. 3.2 mL, *p* = 0.02). The pathophysiological approach has been more frequently used for the hemispheric location (91.3%) compared with the skull base location (91.3% vs. 8.7%) since 2014. The conventional approach was used equally for both the locations (53.3% vs. 46.7%) between 2000 and 2019. The median margin dose was 12 (range: 9–16 Gy), and the median maximal dose was 24 Gy (range: 18–32 Gy). The median prescription isodose line was 50% (range: 37–80%). The treatment marginal dose was significantly lower with the pathophysiological approach than with the conventional approach (median dose: 10 Gy vs. 12 Gy, *p* = 0.01) (Table 1).

### 3.2. Outcome

The median duration of the follow-ups was 3.5 (Range 1–5) years. There were no significant differences in follow-up duration between the two treatment approaches (median follow-up: 3 years vs. 3.6 years, *p* = 0.33).

**Figure 3:**
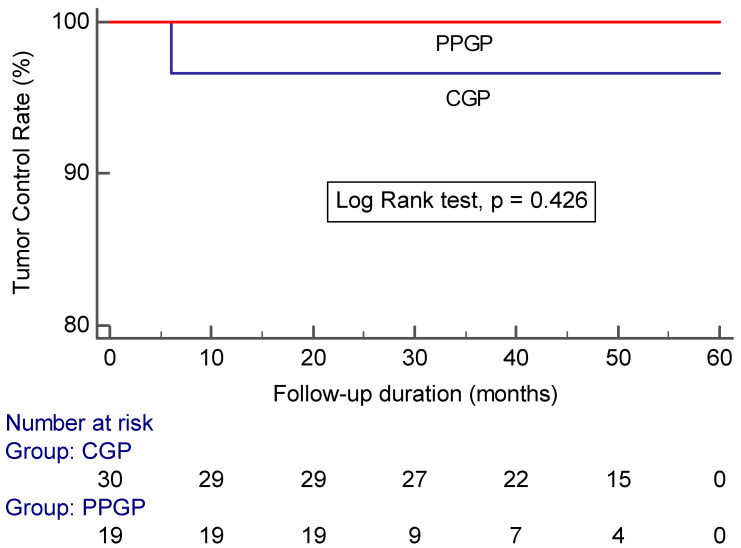
Kaplan–Meier analysis showing tumor control in large incidental meningiomas after GKRS plotted against time in patients treated with a conventional gamma plan vs. pathophysiological gamma plan (CGP: conventional gamma plan; PPGP: pathophysiological gamma plan).

Tumor control was achieved in 52/53 (98.1%) of all incidental meningiomas. Specifically, 16 incidental meningiomas (30.2%) had a volume reduction greater than 50%, 13 (24.5%) had a volume reduction of 15–50%, and 23 (45.3%) were stable in size (volume change <15%) (*p* = 0.816) (Table 2). Tumor control was achieved in 19/19 (100%) incidental meningiomas that underwent a single stage pathophysiological approach compared with 29/30 (96.7%) incidental meningiomas that underwent a conventional approach (log-rank test: *p* = 0.426) (Figure 3).

**Figure 4:**
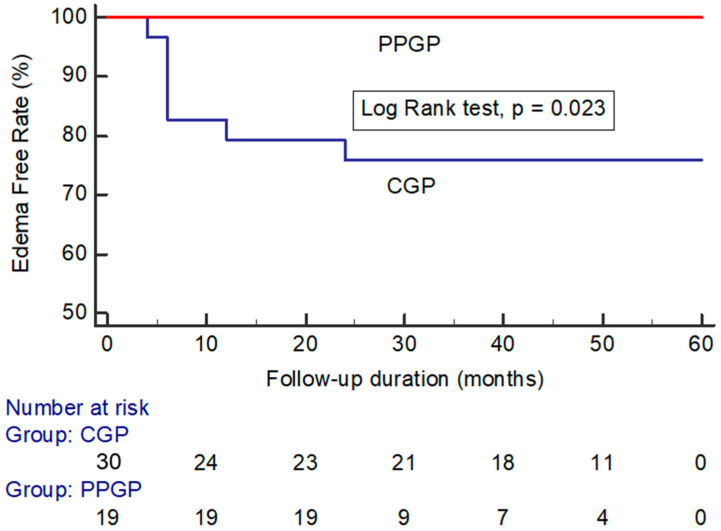
Kaplan–Meier analysis showing peritumoral edema in large incidental meningiomas after GKRS plotted against time in patients treated with a conventional gamma plan vs. pathophysiological gamma plan. (CGP: conventional gamma plan; PPGP: pathophysiological gamma plan).

Peritumoral edema developed in 7/53 (13.2%) of all incidental meningiomas (Table 2). Of the seven incidental meningiomas that developed peritumoral edema, four developed symptomatic peritumoral edema. The maximum value of relative edema indices was 4.55 ± 3.51 at a median time of 12 months (range 6–24 months) after GKRS. The median duration of symptoms was 6 months (range 3–12 months). Most symptoms were relieved with steroids, but one case needed surgery for enlargement and peritumoral edema with new-onset seizure. Surgical pathology showed grade I meningothelial meningioma. Peritumoral edema developed in 0/19 (0%) incidental meningiomas that underwent a single-stage pathophysiological approach compared with 7/30 (23.3%) incidental meningiomas that underwent a conventional approach (log-rank test: *p* = 0.023) (Figure 4).

In incidental hemispheric meningioma, 17 hemispheric meningiomas underwent a single stage pathophysiological approach, while 16 hemispheric meningiomas were treated with a conventional approach. Peritumoral edema developed in 0/17 (0%) incidental hemispheric meningiomas that underwent a single-stage pathophysiological approach compared with 5/16 (31.3%) incidental hemispheric meningiomas that underwent a conventional approach (Fisher’s Exact test: *p* = 0.018).

**Figure 5:**
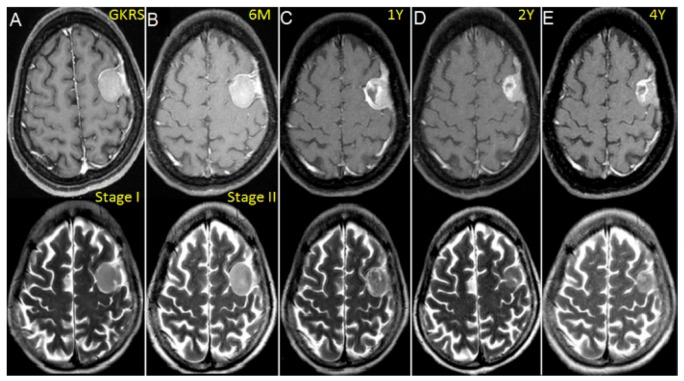
Contrast-enhanced T1 and T2-weighted MR images showing a left-sided convex meningioma (11 mL) treated with volume-staged GKRS in a 45-year-old woman. (**A**) Gamma plan illustrating stage I GKRS with a high margin dose of 18 Gy to the portion of dural attachment of tumor. (**B**) Stage II GKRS with a 10 Gy margin dose for the parenchymal portion of the tumor at 6 months. (**C**) Tumor necrosis noted at 1 year but no evidence of PTE in T2-weighted images. (**D**,**E**) Follow-up images demonstrating that the tumor had markedly shrunken at 2 and 4 years after GKRS.

We observed success with treatment without peritumoral edema even with relatively large incidental meningiomas (>10 mL) using a staged approach 6 months apart (Figure 5).

## 4. Discussion

### 4.1. Tumor Control of Incidental Meningiomas with Stereotactic Radiosurgery

In this retrospective cohort of 53 large incidental meningiomas, the total tumor control was 98.1%. There was no difference in tumor control between a pathophysiological and a conventional approach, although the tumor volume of the pathophysiological approach was relatively larger than that of the conventional approach. A relative lower marginal dose in the pathophysiological approach did not affect tumor control rate.

The efficacy of stereotactic radiosurgery as a primary and an adjuvant therapy for surgically unfavorable small- to medium-sized meningiomas is well-established and shows an equivalent tumor control rate comparable to Simpson grade I resection. According to authors [5,6], because stereotactic radiosurgery is less invasive than surgery and can be performed easily on asymptomatic patients, proactive stereotactic radiosurgery may be beneficial. Tumor control is also high, ranging from 94% to 100%, including our series at 98.6% [2,8,9,18,19].

GKRS can lower the possibility of growth of incidental meningiomas when compared with observation. Jo et al. [2] reported 69 patients who underwent GKRS: tumor size was stable in 57 and decreased in 12 patients, while no patient showed an increase in tumor size (mean follow-up 63.0 months). While tumor volume increased in 24 (31.2%) of 77 patients who initially opted for observation, Kim H et al. [9] revealed that the radiological progression-free survival rates in the GKRS and observation groups were 94.4% and 38.5%, respectively, at 5 years (*p* < 0.001), and 88.5% and 7.9%, respectively, at 10 years (*p* < 0.001). They also reported a volumetric analysis, which showed that untreated tumors gradually increased in size after a long period of observation, and proposed early treatment with stereotactic radiosurgery. A compromised approach including serial monitoring of tumor volumes and regression analysis may reveal the growth pattern of incidental meningiomas and provide information useful for determining a treatment strategy [1]. It has been reported that factors including young age, absence of calcification, peritumoral edema, and high T2 signal intensity are correlated with clinical progression, while calcification is a protective factor [1,2,20,21].

### 4.2. Peritumor Edema Following GKRS

Our series showed the incidence of post-SRS peritumoral edema was 13.5% and symptomatic peritumoral edema was 5.6%, which are comparable to those of previous reports of incidental meningioma, ranging from 8% to 15.3% [8,9,13], but lower than the symptomatic meningioma group at 15.4% to 40% [4,6,7,10,11,12]. The adverse effect of peritumoral edema all occurred in the conventional approach group, but none took place in the pathophysiological approach. There was a significant reduction in peritumoral edema associated with the pathophysiological approach compared with the conventional approach (*p* = 0.01). The pathophysiological approach applied a relative lower median margin dose 10 (10–14) Gy. to treat tumor margins neighboring the cortical surface, which was lower than the dose of edematous patient group 14 (12–16) Gy. Similar reports published on symptomatic meningioma revealed a higher prescription dose (>15 Gy to 16 Gy) is one of the risk factors of edema development after stereotactic radiosurgery.

Tumor size is another factor; in our experience, peritumoral edema seldom occurs in small tumors, so we only included relative larger tumors. Ide et al. [15] observed the histology of meningioma reported when a tumor is small: the intact leptomeninges and brain cortex prevent the easy spreading of vasogenic edema fluid to the white matter. Peritumoral edema usually occurred in larger tumors, and other reports for incidental meningioma also had similar findings. Hoe Y et al. [8] reported tumor volume >4.2 mL; our study showed tumors larger than 2 mL had high risk for peritumoral edema (13.5%). For the symptomatic meningioma group, almost all cases demonstrated that larger volume is a significant factor [4,6,10,11,12,13]. A larger tumor damages the leptomeninges and brain cortex and comes into direct contact with the white matter, allowing direct transmission of the humoral edema-promoting factor or edema fluid into the white matter, resulting in vasogenic edema [15,16]. This explains that a pathophysiological approach with a lower marginal dose for the parenchymal site reduces peritumoral edema. In our study, although the tumor size was relatively larger in the pathophysiological approach group, the peritumor edema occurred less than in tumors in the conventional approach group.

It is important to consider these results within the context of the meningioma location. Previous reports showed skull base meningioma constitutes a different clinical and biologic disease entity, and the absence of a significant prognostic effect is most likely due to skull-base tumors comprising a minority of truly asymptomatic meningiomas [22]. In incidental hemispheric meningioma, the results showed a significant reduction in peritumoral edema associated with the pathophysiological approach compared with the conventional approach. The incidence of peritumor edema was relatively lower, which may be explained with this different approach.

Corticosteroids are most commonly used to reduce the peritumoral edema associated with meningiomas. Most of our patients who later developed symptomatic peritumoral edema could be controlled with steroids. The use of prophylactic anticonvulsants may also be considered depending upon the degree and the location of the edema. One of our symptomatic edema patients had worsening edema and seizure attacks, so later, he underwent resection of a meningioma. However, it is rare for a meningioma treated with stereotactic radiosurgery to necessitate resection for brain edema control alone, as this edema is transient and is usually manageable with medical therapy. Some articles also reported that vascular endothelial growth factor inhibitors, such as bevacizumab, have been shown to reduce baseline peritumoral edema as well as edema following radiosurgery and radiation therapy [23,24].

### 4.3. Rationale of Pathophysiological Approach for Relative Large Incidental Meningioma Treated by Stereotactic Radiosurgery

The basis of our pathophysiological approach, targeting the feeding artery with a higher dose and the parenchyma with lower dose, can be found within existing literature regarding radiation treatment mechanisms and the pathophysiology of meningiomas. First, existing literature supports the use of lower doses to reduce radiation side effects and have suggested that it is an option for meningioma treatment [25,26,27,28,29]. Radiation-induced apoptosis is thought to explain the efficacy of low-dose GKRS [26,30]. Tsuzuki et al. reported a large proportion of proliferation cells may be susceptible to the induction of apoptosis, and even some tumors with the presence of Bel-2 might not suppress this gamma knife effect [30]. Other reports also revealed it is feasible to use lower doses to the tumor margin and the volume stage to reduce radiation side effects [14,20,28,31,32].

Second, we also used a larger dose for the dural side, causing feeding vessels in the radiation field to become progressively dysfunctional, which led to to the ischemic death of the tumor [33,34]. The radiobiological effect of GKRS on meningioma is a combination of both a cytotoxic effect and a delayed vascular effect. Pathological examination demonstrated irradiation induces vasculature stenosis, occlusion, and tumor degeneration as a result of reduced blood supply [35]. Because the meningioma arises from the dura mater, most tumors are predominantly supplied by meningeal vessels; therefore, it is reasonable to target the dural feeding artery site with a higher dose to enhance the tumor control and the parenchymal margin of the tumor with a lower dose to reduce peritumoral edema.

The result of our pathophysiological approach was high tumor control despite treating larger tumor volumes with lower doses and, therefore, reduced peritumoral edema even with relatively large incidental meningiomas. Peritumoral edema was present in 13.2% (*n* = 7) of all cases and comparable to rates of peritumoral edema reported in the literature on incidental meningioma ranging from 9% to 15.3% [8,9,13]. All cases of peritumoral edema were found with the conventional approach, compared with none using the pathophysiological approach. It is worth noting that over 90% of our tumors treated with the pathophysiological approach were also in the hemispheric location, a factor associated with increased peritumoral edema.

### 4.4. Limitation and Extension of GKRS for Incidental Meningiomas

Our study has potential limitations that may confound the interpretation of our findings. First, we did not have histology reports available to accurately grade incidental meningioma, which is also known to affect treatment outcomes. Though overall, past publications have reported that more than 80% are grade I benign tumors [36], while atypical grade II includes 4–15% of meningiomas, and malignant grade III accounts for 1–3% of cases [17,37] In the 2004–2010 U.S. cohort of primary brain tumors, the proportion of each grade was 94.6%, 4.2%, and 1.2%, respectively [38]. Islim et al. [17] reported a surgical group for 316 patients, 303 of which had WHO grade I meningioma 95.8%, 10 (3.16%) had WHO grade II meningioma, while in 3 (0.95%), the pathology revealed WHO grade III meningioma. Regular follow-up is important, as results of 4–5% of grade II-III meningioma may not be ordinarily predictable. Second, we did not pursue additional adjustments in our analysis of other risk factors such as tumor location and calcification given the limited numbers, though we did note that the hemispheric location predominated among meningiomas treated with a pathophysiological approach. Finally, our cohort size is still modest, and further prospective larger-scale studies will be needed to examine the external validity of our findings.

## 5. Conclusions

GKRS for incidental meningiomas achieves a good tumor control rate for incidental meningioma. A pathophysiological approach targeting the dural feeding artery with a higher dose and the parenchyma with a lower dose results in effective tumor control with reduced peritumoral edema among incidental meningiomas ≥ 2 cm in diameter and/or ≥2 mL in volume treated with GKRS. A two-staged approach may be considered for relatively large tumors > 10 mL. A pathophysiological approach may be a reasonable alternative to reduce the risk of peritumoral edema when treating large incidental meningiomas with GKRS.

## Figures and Tables

**Table 1:** Baseline characteristics by treatment approach ^a^.

	All Tumors (*n* = 53)	Pathophysiological Approach (*n* = 23)	Conventional Approach (*n* = 30)	*p*-Value
Patient age (years)	64 (33–87)	68 (45–87)	61 (33–78)	0.12
Female/Male	46 (86.8%)/7 (13.2%)	18 (78.3%)/5 (21.7%)	28 (93.3%)/2 (6.7%)	0.22
Volume (mL) ^b^	3.5 (2–27.2)	5.3 (2.2–27.2)	3.2 (2–15)	0.02
Location				0.003
Hemispheric	37 (69.8%)	21 (91.3%)	16 (53.3%)	
Skull base	16 (30.2%)	2 (8.7%)	14 (46.7%)	
Marginal Dose (Gy) ^b^	12 (9–16)	10 (10–14)	12 (9–16)	0.01

^a^ Data are presented as median (range). ^b^ Abbreviations: mL = milliliter; Gy = Gray.

**Table 2:** Outcomes by treatment approach.

	All Tumors (*n* = 53)	Pathophysiological Approach (*n* = 23)	Conventional Approach (*n* = 30)	*p*-Value
Follow-up (years)	3.5 (1–5)	3 (1–5)	3.6 (1–5)	0.33
Tumor control	52 (98.1%)	23 (100%)	29 (96.7%)	1.00
Volume change				0.82
>75% decrease	1 (1.9%)	1 (4.3%)	0 (0%)	
50–75% decrease	15 (28.3%)	7 (30.4%)	8 (26.7%)	
15–49% decrease	13 (24.5%)	5 (21.7%)	8 (26.7%)	
<15% change	23 (43.4%)	10 (43.5%)	13 (43.3%)	
>15% increase	1 (1.9%)	0 (0%)	1 (3.3%)	
Peritumoral edema	7 (13.5%)	0 (0%)	7 (24.1%)	0.01

Data are presented as median (range).

## Data Availability

The data that support the findings of this study are available from the corresponding author upon reasonable request.
